# The Impact of Electron Correlation on Describing QM/MM Interactions in the Attendant Molecular Dynamics Simulations of CO in Myoglobin

**DOI:** 10.1038/s41598-020-65475-2

**Published:** 2020-05-22

**Authors:** Xianwei Wang, Chenhui Lu, Maoyou Yang

**Affiliations:** 1 College of Science, Zhejiang University of Technology, Hangzhou Zhejiang, 310023 China; 2 College of Mechanical Engineering, Shanghai University of Engineering Science, Shanghai, 201620 China; 3 School of Electronic and information Engineering (Department of Physics), Qilu University of Technology (Shandong Academy of Sciences), Jinan, Shandong 250353 China; 4 State Key Laboratory of Precision Spectroscopy, East China Normal University, Shanghai, 200062 China

**Keywords:** Computational chemistry, Biological physics

## Abstract

The impact of the dispersion and electron correlation effects on describing quantum mechanics/molecular mechanics (QM/MM) interactions in QM/MM molecular dynamics (MD) simulations was explored by performing a series of up to 2 ns QM/MM MD simulations on the B states of the myoglobin–carbon monoxide (MbCO) system. The results indicate that both dispersion and electron correlations play significant roles in the simulation of the ratios of two B states (B_1_/B_2_), which suggests that the inclusion of the electron correlation effects is essential for accurately modeling the interactions between QM and MM subsystems. We found that the QM/MM interaction energies between the CO and the surroundings statistically present a linear correlation with the electric fields along the CO bond. This indicates that QM/MM interactions can be described by a simple physical model of a dipole with constant moment under the action of the electric fields. The treatment provides us with an accurate and effective approach to account for the electron correlation effects in QM/MM MD simulations.

## Introduction

Mixed multiscale QM/MM^1–5^ models are routinely utilized in studies exploring the structure, reactivity, and electronic properties of proteins and other biological molecules^6–11^. In such models, the part of a large system that is of primary interest (e.g., the active site of an enzyme) is treated using an electronic structure method for capturing more accurate interaction energies, with the surroundings treated using an MM approach. Generally, the current QM/MM approaches use a single hybrid Hamiltonian to describe the system:

1 $${\rm{H}}={{\rm{H}}}_{{\rm{QM}}}+{{\rm{H}}}_{{\rm{MM}}}+{{\rm{H}}}_{{\rm{QM}}/{\rm{MM}}}$$

where ${{\rm{H}}}_{{\rm{QM}}}$ is the Hamiltonian describing the QM subsystem, ${{\rm{H}}}_{{\rm{MM}}}$ is the MM Hamiltonian, and ${{\rm{H}}}_{{\rm{QM}}/{\rm{MM}}}$ is the coupling term in the Hamiltonian describing the QM and MM interactions. Under this representation, the total energy of a given system can be obtained from the computation of the lowest eigenvalue of the Hamiltonian in Eq. 1.

2 $$E={E}_{{\rm{QM}}}+{E}_{{\rm{MM}}}+{E}_{{\rm{QM}}/{\rm{MM}}}$$

The QM/MM formulation is flexible enough to accommodate almost all types of QM methods (e.g., semi-empirical methods, density functional theory (DFT)-based QM methods and *ab initio* (wave function (WF)-based) methods) in combination with an MM method (Amber, CHARMM, or a number of polarizable force fields)^12–15^.

Although the QM/MM treatment of the interactions is not exactly the same as that of the QM method, the change in the interactions (e.g., during a reaction or the dynamics of a biomolecular hydrogen bond) are modeled well by the QM/MM method using the electrostatic embedding approach^16^. This is as expected, because most of the protein functions are primarily achieved by the electrostatic^17–21^. For example, as previous works demonstrate, the electric field that a protease exerts at the active site is one of the most important origins of its catalytic power^22–26^.

With the advances in computer hardware and computational methods^27–31^, a number of QM/MM molecular dynamics (MD) studies employing various implementations of the expensive (prohibitive time/computational costs) Møller–Plesset perturbation theory (MP2) have been conducted^32–43^. However, it remains challenging to apply potentially highly accurate correlated *ab initio* QM methods (e.g., coupled clustering) in QM/MM MD simulations with a large number of QM atoms. Therefore, semi-empirical, DFT, or even Hatree–Fock (HF) methods have been employed in most QM/MM studies. However, DFT and HF methods suffer from a well-known limitation; that is, the Coulomb correlation is missing in the mean-field HF approach, which results in its inability to account for the dispersion effects, whereas many DFT-based QM methods cannot account for these effects in precise terms because the exact correlation functional is unknown^44,45^. Consequently, DFT methods do not always provide correct results in QM/MM simulations^46–49^, whereas HF method can only work well in the QM/MM simulation of specific systems (e.g., methyl-transfer reactions)^50^. Great efforts have been made to account for the dispersion effects in QM methods over the past decade, with a number of empirical dispersion methods routinely used for the QM calculations^45,51–53^.

The inclusion of the electron correlation and dispersion effects is mandatory for QM studies related to chemical systems and various intramolecular phenomena^27,44^. Investigation of the importance of these effects on the accurate description of the QM/MM interactions is a primary issue for realistic MD simulations of large or condensed systems with a QM/MM protocol. For this, first, an appropriate model system is required. Such a system should have a characteristic quantity (e.g., reaction barriers) that is easily handled by the QM/MM simulation with a small-sized QM region to facilitate the convergence of the simulation, and the characteristic quantity should be sensitive to the electrostatic interactions. It is preferable not to have dangling bonds between the QM and MM subsystems to avoid introducing unnecessary effects from the QM/MM boundary^54^.

**Figure 1:**
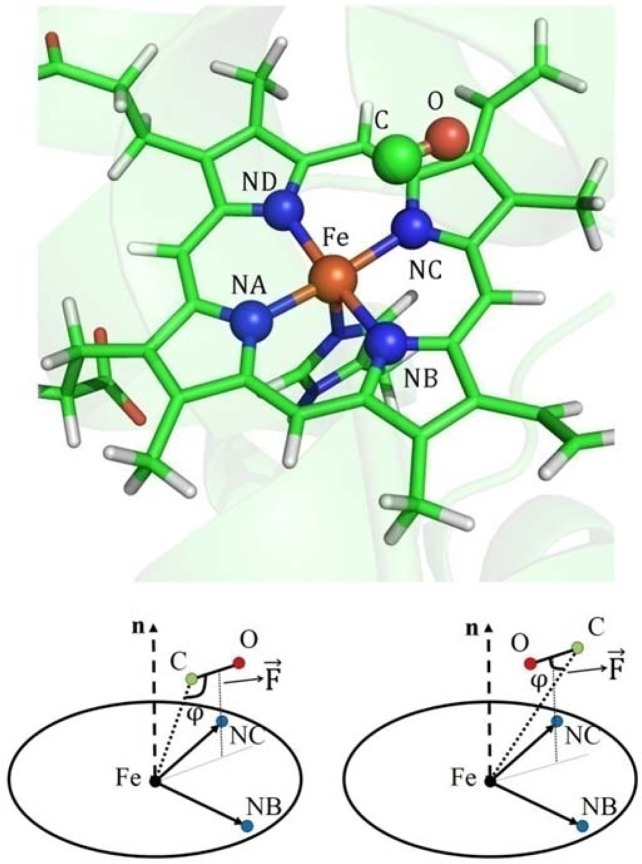
The location of the CO in the docking site of the myoglobin for the B state (upper). The structure was extracted from the QM/MM MD simulation. Graphical representations of the B_1_ state (lower left panel) and B_2_ state (lower right panel). Here, ${\rm{\varphi }}$ is the angle that is defined as Fe $\cdots$C$\equiv$O. The heme is depicted as a circular plate, and n is the normal vector of the plate plane, and $\overrightarrow{F}$ represents the electric field exerted by the environment.

The MbCO system is one of the most studied proteins for investigating ligand binding, migration or other biologically relevant processes^55–74^. The spectroscopic characterization of CO has been used to explore the relationship between the dynamics, structure, and function of proteins in numerous experimental studies^63,71,73–75^. The molecular migration pathway for the CO to leave the myoglobin involves a set of transition steps between certain specific cavities, including the so-called distal heme pocket (the “docking” site) and four sites (Xe1–4) identified by the host xenon^75–77^. The CO binds itself to the myoglobin by forming a covalent bond with the center iron (Fe) of the heme before it then moves to the docking site adjacent to the heme at the edge of the distal pocket following photodissociation^62,68,70,78,79^. A previous study^75^ from Alben *et al*. demonstrated that the features of the time-resolved polarized absorbance spectra of the CO in the bound and docking sites of myoglobin are different and were accordingly labeled as states A and B, respectively. Meanwhile, further splitting of the infrared spectrum with two well-resolved bands of photodissociated CO in the docking site (B state) was observed by Lim *et al*.^71^. Previous theoretical studies^78–81^ have demonstrated that the splitting is a result of two different orientations of CO (B_1_ and B_2_, which correspond to high frequency and low frequency, respectively) within the same site of the distal heme pocket with the carbon or oxygen atom closer to the center Fe of the heme. The relative locations of the CO and the heme for the B_1_ state and a graphical representation of the B_1_ and B_2_ states are shown in Fig. 1. In our previous study^80^, we demonstrated that the obtuse angle of Fe$\cdots$C$\equiv$O (labeled as ${\rm{\varphi }}$ in Fig. 1) with the most likely population of around 115° corresponded with the B_1_ state, whereas the acute angle of ${\rm{\varphi }}$ with the most likely population of around 50° corresponded with the B_2_ state. Meanwhile, Lim *et al*. demonstrated that the ratio of the two states (B_1_/B_2_) was not 1:1 but ~1.7:1^72^, with the ratio sensitive to the electrostatic interactions (including the polarization effect and the multipole electrostatic interactions) between the CO and its surroundings, which has been confirmed in numerous theoretical studies^78–80^. Therefore, the MbCO system is a particularly striking model for exploring the role of electron correlation effects in QM/MM MD simulations.

The HF method is a mean-field electronic structure method. The calculated molecular dipole moment using such an approach would be overestimated because of the failure to consider the electron correlation effects^82^. DFT-based methods such as the B3LYP are often expected to provide a more accurate description of the electrostatic interactions because they account for the electron correlation in their theoretical basis^83^. However, B3LYP functionals cannot provide a sufficient description of the dispersion^44,45^. Although the WF-based singles and doubles coupled-cluster method with perturbative triples (CCSD(T)) is regarded as the current gold standard in quantum chemistry, it remains impractical to utilize such a method in QM/MM MD simulations due to the huge computational costs. The MP2 offers a good combination of accuracy and low computational costs and has the capacity to provide a good estimation of the correlation energies. In practice, the MP2 has been used as benchmark calculations in dispersion-dominated complexes^84,85^.

In this study, a series of up to 2 ns QM/MM MD simulations using HF, B3LYP, and MP2 methods were performed in view of investigating the impact of electron correlations on the dynamics of the CO in the docking site of myoglobin. The physical fundamental of the MbCO system possesses two B states with different distribution ratios, and the correlations between the QM/MM electrostatic interactions and the electric fields are thus explored. We also discuss how a better description of the QM/MM interactions can be achieved.

## Methods

### MD simulations

The A state of the MbCO structure (PDB id: 1MBC^86^), in which the CO binds itself to the center Fe of the heme, was used as the initial simulation model. The LEaP module^87^ of the Amber program was utilized to add the missing hydrogen atoms. The amine groups of all lysine (Lys) and arginine (Arg) residues were protonated, whereas the carboxylic were deprotonated for all aspartic acid (Asp) and glutamic acid (Glu) residues. All histidine (His) residues were left neutral and protonated at the N$\delta 1$ position based on the local electrostatic environment, with the exception of His93, which was protonated at the N${\rm{\varepsilon }}2$ position. Meanwhile, the N-terminal was protonated, whereas the C-terminal was deprotonated. The force field parameter given by Giammona^88^ was used to simulate the heme and the CO. The protein was placed in a periodic truncated octahedron boxer of TIP3P water molecules. The closest distance between the surface of the boxer and the protein atom was set to 12 Å. Three minimization steps based on the Amber ff99SB force field and short MD simulations were employed to relax the system and bring the CO molecule from the bound site (A state) to the docking site (B state). The bonding interaction between the CO and the center Fe of the heme was removed so that the CO would be dissociated under the action of the repulsive term of the van der Waals interactions. First, the solvent molecules were optimized with all protein atoms constrained to their initial structure. Second, the backbone protein atoms and all the heme and CO atoms were constrained, and the other protein atoms and the solvent molecules were relaxed. Third, all the protein and solvent atoms were energy minimized, and the heme and the CO were still constrained to their initial structure. Then, the system was heated to 300 K at 100 ps with all the heme and CO atoms constrained. Following this, a 100-ps MD simulation without constraint was carried out to simulate the photodissociation with a periodic boundary condition at 300 K and 1 atm. The time integration step was set to 2.0 fs with the application of the SHAKE^89^ algorithm to maintain all hydrogen atoms in reasonable positions. The particle-mesh Ewald method^90^ was applied to treat long-range electrostatic interactions. A 10 Å cut-off was implemented for the van der Waals interactions. Langevin dynamics^91^ was used to regulate the temperature with a collision frequency of 1.0 ps^−1^.

A series of up to 2 ns QM/MM MD simulations was independently performed following the classical equilibrium simulation. Only the CO was partitioned into the QM subsystem, whereas the remainder of the system, including the heme, protein, and solvent was partitioned into the MM subsystem. Corresponding force field parameters that were similar to the classical equilibrium simulation were used for the MM subsystem. The QM subsystem was treated by the B3LYP hybrid density functional with various basis sets of 6–31 G, 6–31 G**, 6–31++G**, aug-cc-pVDZ and aug-cc-pVTZ in the QM/MM MD simulations to test the effect of the size of the basis sets. The other two MD simulations were performed at the HF/aug-cc-pVTZ/amber99SB and MP2/aug-cc-pVTZ/amber99SB QM/MM levels. In all QM/MM MD simulations, a 25 Å cutoff was utilized to treat QM/MM electrostatic interactions. The electronic coupling between the QM and MM subsystems was treated by including MM charges in the QM Hamiltonian. The integration step was set to 1.0 fs and trajectories were generated with the structures saved every 200 fs. The angles of the Fe$\cdots$C$\equiv$O were calculated using the saved structures (1 × 10^4^ configurations in total) and were subsequently used to analyze the ratios of the two B states. The Amber12 program^87^ was used to perform the MD simulations. The sander module with an interface to the Gaussian09 program^92^ was utilized to perform the QM/MM MD simulations.

### Calculation of the electric field and QM/MM interactions

The electric field along the CO bond and the electrostatic interaction energies between the QM and MM subsystems were calculated and applied for exploring the foundation of that how the inclusion of the electron correlation and dispersion effects influences QM/MM MD simulations. Although the QM/MM MD simulations at various QM/MM levels generated a series of trajectories, the calculations of the electric field and the interaction energies were performed on the basis of only one of them to test the correlations between the relatively independent calculations and the simulated results. The obtained (B_1_/B_2_) ratio is ~1.9:1 based on the MD simulation at the B3LYP/6–31 G** QM/MM level. This ratio is somewhat modest for studying both the B_1_ and B_2_ states. Therefore, the QM/MM interactions were calculated using the HF, B3LYP and MP2 QM methods combined with the aug-cc-pVTZ basis set based on this trajectory and the calculated results were plotted as the function of the calculated electric fields, and then compared with the QM/MM simulated results (the distribution of the angle of the Fe$\cdots$C$\equiv$O) at the HF/aug-cc-pVTZ, B3LYP/ aug-cc-pVTZ and MP2/ aug-cc-pVTZ QM/MM levels.

### Calculation of the electric field

A total of 10,000 configurations generated from the 2 ns QM/MM MD simulations were used to calculate the electric fields along the CO bond using the charge model of the corresponding MM force field. Here, the electrostatic potential at the center of the C and O atoms of the CO molecule was first calculated as follows,

3 $$\varphi (\overrightarrow{r})=\frac{1}{4\pi {\varepsilon }_{{\rm{eff}}}}\sum _{i}\sum _{j\epsilon i}\frac{{q}_{ij}}{|\overrightarrow{r}-{\overrightarrow{r}}_{ij}|}$$

where *i* denotes the residue number that runs over all the residues of the protein, heme and solvent molecules (6,855 waters); *j* is the atom number in residue *i*; ${q}_{ij}$ and ${\overrightarrow{r}}_{ij}$ are the atomic charge and position vector of atom *j* respectively; $\overrightarrow{r}$ is the position vector C or O atoms of the CO molecule; ${\varepsilon }_{{\rm{eff}}}$ denotes the effective dielectric constant and was set to 1.0 since the explicit water model was utilized^93^.

The mean electric field along the CO bond was obtained using the following expression,

4 $$\overrightarrow{{\rm{F}}}({\overrightarrow{r}}_{{\rm{CO}}})=\frac{\varphi ({\overrightarrow{r}}_{{\rm{C}}})-\varphi ({\overrightarrow{r}}_{{\rm{O}}})}{|{\overrightarrow{r}}_{{\rm{CO}}}|}$$

where $\varphi ({\overrightarrow{r}}_{{\rm{C}}})$ and $\varphi ({\overrightarrow{r}}_{{\rm{O}}})$ are the electrostatic potentials at the center of the C and O atoms of the CO, which were calculated using Eq. 3, and $|{\overrightarrow{r}}_{{\rm{CO}}}|$ is the bond length of the CO.

### Calculation of the electrostatic interactions

Two schemes were utilized to calculate the electrostatic interaction energies between the QM (CO molecule) and MM subsystems (the surroundings). The first was based on the point charge model, whereas the ESP charges derived from the QM/MM calculations were used for the CO to ensure the polarization effect was taken into account. The charges of the MM subsystem were taken from the corresponding force field parameter. One limitation of this scheme is that it does not account for the electrostatic multipole interactions. The second scheme was based on the QM/MM calculations. Here, a QM energy was first obtained from the QM/MM calculations of the QM subsystem in the protein environment. The obtained energy (labeled as ${E}_{{\rm{total}}}$) contained the self-energy of the QM subsystem (labeled as ${E}_{{\rm{self}}}$) and the electrostatic interactions between the QM and MM subsystems (labeled as ${E}_{{\rm{EI}}}$). The ${E}_{{\rm{self}}}$ of the QM subsystem could be obtained from the QM calculations in gas phase, and the ${E}_{{\rm{EI}}}$ could then be obtained by deducting the ${E}_{{\rm{self}}}$ from the ${E}_{{\rm{total}}}$. The calculated interaction energies were plotted as a function of the electric fields to provide a statistics analysis of the foundation of the dynamic behavior of CO in the docking site of myoglobin and to allow us to explore the correlation relationship between the QM/MM interaction energies and the electric fields.

## Results and Discussion

**Figure 2:**
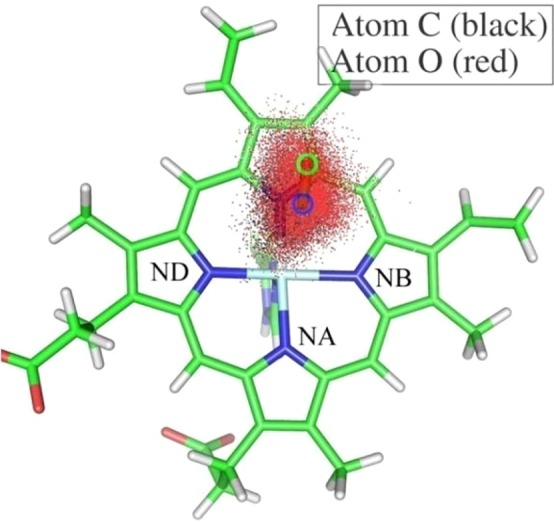
Contour plots of the probability distribution of the C and O atoms of the CO molecule on the heme plane during one of the trajectories generated by the 2 ns QM/MM MD simulation. The relative locations of the B_1_ state of the CO obtained from the X-ray experiment^95^ are marked by the blue circle (C atom) and the green circle (O atom).

In previous works^78,94^, it was observed that photodissociated CO only remains in the docking site for nanoseconds before it then rapidly moves to other regions of the protein. To confirm that the CO molecule was located in the docking site of the myoglobin in all 2 ns MD simulations, contour plots presenting the cumulative distribution projected onto the heme plane of the C and O atoms during the 2 ns QM/MM MD simulation were created as shown in Fig. 2, and an X-ray experimental structure^95^ of the B_1_ state is also shown in Fig. 2. It can be seen from the contour plot that the distributions of the C and O atoms were both concentrated. The predicted locations of the B state in the 2 ns QM/MM MD simulation were at the edge of the distal heme pocket adjacent to the porphyrin ring *c*, which is in good agreement with the X-ray experiment. Meanwhile, the CO molecule lay approximately parallel to the heme plane in the 2 ns QM/MM MD simulation, which is also in good agreement with the X-ray experiment. More detailed analysis indicated that the distribution of the center of mass of the CO for both the B_1_ and the B_2_ states was concentrated in the same location, with the CO molecule displaying an antiparallel orientation. The C and O atoms of the CO alternately formed a weak hydrogen bond with the H atom of the −NH group of the His64 during the MD simulations, which provided an anchoring force for trapping the CO. In fact, the CO molecule remained in the docking site following 4 ns QM/MM MD simulations. Therefore, 2 ns MD simulations will not make the CO molecule move out of the docking site.

**Figure 3:**
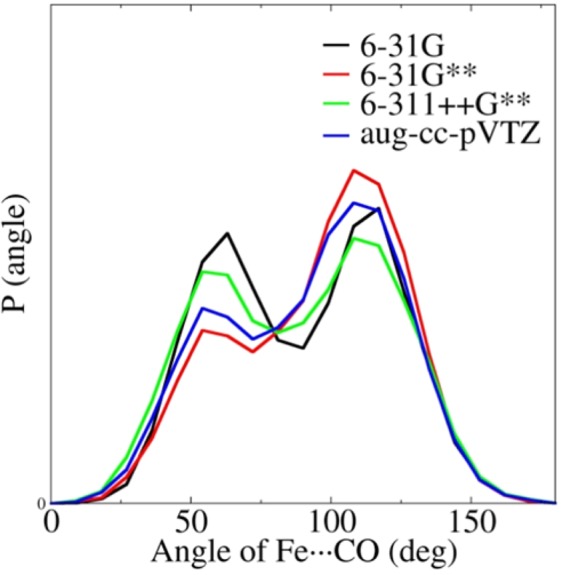
The distribution of the Fe$\cdots$C$\equiv$O angle for the QM/MM MD simulations at the B3LYP with various basis sets of 6–31 G, 6–31 G**, 6–311++G**, and aug-cc-pVTZ QM/MM levels.

The B3LYP is one of the most widely used density functionals^44^ and is expected to provide an accurate calculation of the electrostatic potentials for protein. The QM/MM MD simulation was thus first performed using the B3LYP method. To test the effects of the size of the basis sets, a series of up to 2 ns QM/MM MD simulations with a QM subsystem treated via B3LYP/6–31 G, B3LYP/6–31 G**, B3LYP/6–311++G**, and B3LYP/aug-cc-pVTZ were carried out, with the results shown in Fig. 3. It can be seen from the figure that the size of the basis sets in the QM/MM MD simulations had an obvious effect on the simulated results. The predicted ratio of the B_1_ (corresponding to the ~115° angle of the Fe$\cdots$C$\equiv$O) and B_2_ (corresponding to the ~55° angle of the Fe$\cdots$C$\equiv$O) states using the B3LYP/6–31 G QM method was ~1.1:1, and the proportion of the B_1_ state enhanced the ratio, which became ~1.8:1 when the polarization function was introduced (6–31 G** basis set). The results demonstrate that the introduction of the polarization function in the basis sets had a significant impact on the simulations using the QM/MM method. However, the ratio fell back to ~1.1:1 when the diffuse function was added (the result of 6–311++G** in Fig. 3), which indicates that the diffuse function and the polarization function have different roles in impacting the QM/MM simulations. The ratios of the two B states obtained via the B3LYP/aug-cc-pVDZ and B3LYP/aug-cc-pVTZ methods were almost the same (see Figure S1), which indicates that the further improvement in the basis sets after the basis set of aug-cc-pVDZ was achieved had very little influence on the simulations. The predicted ratio of the two B states was ~1.5:1 using the B3LYP/aug-cc-pVTZ method, which was closest to that using the B3LYP/6–31 G**, meaning the basis set of 6–31 G** may provide a good balance in terms of accuracy and speed due to the error cancellation.

**Figure 4:**
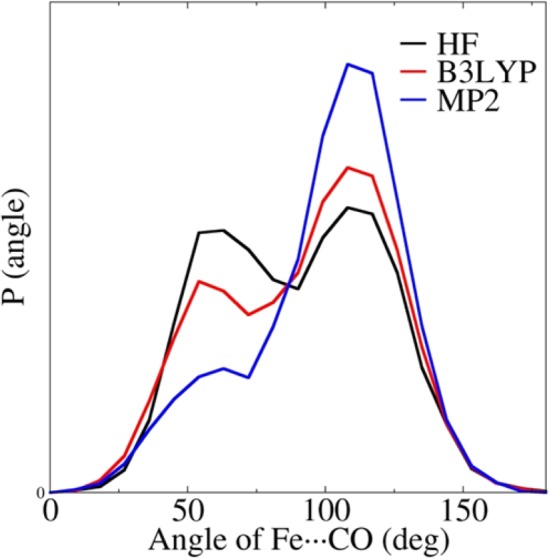
The distribution of the Fe$\cdots$C$\equiv$O angle for the QM/MM MD simulations at the HF/aug-cc-pVTZ/Amberff99SB, B3LYP/aug-cc-pVTZ/Amberff99SB, and MP2/aug-cc-pVTZ/Amberff99SB QM/MM levels.

Although the density functional B3LYP is regarded as providing a more accurate description of molecular electrostatic potentials than the HF method due to the consideration of certain electron correlation effects, the B3LYP does not account for the dispersion. Thus, to explore the role of dispersion and electron correlation in QM/MM MD simulations, two 2 ns QM/MM MD simulations of MbCO with a QM subsystem treated via HF/aug-cc-pVTZ and MP2/aug-cc-pVTZ were performed. The obtained distribution of the Fe$\cdots$C$\equiv$O angles is plotted in Fig. 4. For comparison, the results based on the B3LYP/aug-cc-pVTZ/Amberff99SB QM/MM MD simulation were also plotted. It can be seen from Fig. 4 that there was a significant difference in the ratios of the two B states predicted by the HF/aug-cc-pVTZ/Amberff99SB, B3LYP/aug-cc-pVTZ/Amberff99SB, and MP2/aug-cc-pVTZ/Amberff99SB QM/MM MD simulations. In terms of the results obtained from the HF/aug-cc-pVTZ/Amberff99SB QM/MM MD simulation, the B_1_/B_2_ ratio increased from ~1.1:1 to ~1.5:1 for the simulation at the B3LYP/aug-cc-pVTZ/Amberff99SB QM/MM level, which indicates that the inclusion of the electron correlation is essential in QM/MM MD simulations. The B_1_/B_2_ ratio calculated from the MP2/aug-cc-pVTZ/Amberff99SB QM/MM MD simulation was ~3.5:1, a more than threefold increase on that obtained via the HF method and a more than twofold increase on that obtained via the B3LYP method. The obtained ratio at the MP2/aug-cc-pVTZ/Amberff99SB QM/MM level demonstrated a more significant change in that at the B3LYP/aug-cc-pVTZ/Amberff99SB QM/MM level compared with the result of the HF/aug-cc-pVTZ/Amberff99SB QM/MM simulation. Therefore, it can be stated that it is essential to adopt a QM method that accounts for the dispersion effect in QM/MM MD simulations to obtain accurate results. However, in most QM/MM MD simulations, dozens or even hundreds of atoms have to be partitioned into a QM subsystem. Although correlated WF methods (such as MP2 or CCSD(T)) used in combination with a moderate-sized basis set, such as a aug-cc-pVTZ, could provide accurate results for the simulations, it remains impractical to obtain convergent results using these methods due to the huge computational cost. It is thus essential to make efforts to develop accurate and effective methods that account for the electron correlation effects in QM/MM MD simulations.

It is worth noting that the results based on the B3LYP/aug-cc-pVTZ/Amberff99SB QM/MM model had the best agreement with the experimental measurement of 1.7:1, but this does not prove that the B3LYP performs better than the MP2 for QM/MM MD simulations. In fact, it may indicate that a larger QM region is required for a more accurate simulation of the ratio of the two B states and that the QM region should include the heme, given the strong QM interactions between the center Fe of the heme and the CO molecule. To accurately model the QM interactions, a new MM method may need to be developed. However, it is important to note that the aim of this work was to explore how the QM/MM interactions can be accurately modeled. Here the MbCO system served as a model system rather than a real system, meaning we simply focused on the differences in the simulated results of various QM/MM models.

Thus far, we have examined the role of electron correlation in the QM/MM simulations of the dynamics of the CO molecule in the docking site of myoglobin and have indicated its significance for determining the ratio of the two B states. Next, the theoretical fundamental of possessing two MbCO B states is explored. In previous works^22,74,78^, it was indicated that the two B states are stabilized by the electrostatic interactions between the CO and its surroundings and that the splitting of the infrared spectrum is derived from the Stark effect where the photodissociated CO in the same docking site experiences the electric field with antiparallel orientations. The conclusions suggest that there may be a specific relationship between the electrostatic interactions and the electric fields. An investigation of this relationship could help us to better understand the dynamics of the CO in the docking site of myoglobin and could reveal new physical insights into molecular interactions.

**Figure 5:**
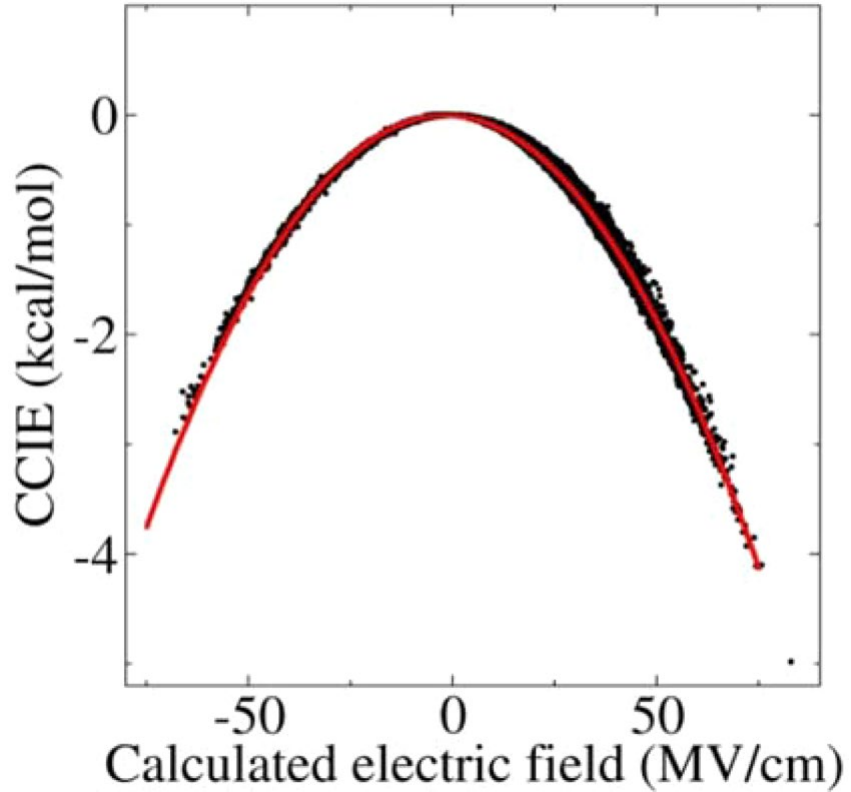
Distribution of the CCIEs between the QM (CO molecule) and the MM (the surroundings) as a function of the electric fields along the CO bond, which exhibits a quadratic function relationship. The influence on the CCIEs of the changes in the bond length of the CO was eliminated.

The classical electrostatic interactions (calculated on the basis of the point charge model) as a function of the electric fields are shown in Fig. 5. It can be seen from the figure that the classical electrostatic interactions present an exact quadratic function relationship with the electric fields and that the quadratic function relationship has a y-axis (vertical axis) symmetry. Although the electrostatic energies presented in Fig. 5 were mainly derived from the polarization effect, in reality, the calculated electrostatic interactions were also influenced by the change in bond length of the CO, with the exception of the polarization effect of the electric field. Meanwhile, the influence on the calculated interaction energies from the change in the bond length of the CO was small and relatively independent from that of the polarization effect. Therefore, the quadratic function relationship remained true and exhibited a y-axis (vertical axis) symmetry, even given the effect of the change in the bond length of the CO (see Figure S2), whereas the zero point of the quadratic function was almost unchanged because the ESP charge of the CO in the gas phase calculated at the B3LYP/aug-cc-pVTZ QM level was very small. The distribution of the classical interaction energies was symmetrical in the positive and negative electric fields, meaning it should not result in a different ratio for the two B states. As such, it is not a primary factor in the B state of the MbCO system possessing a different population, which is consistent with previous research^78,79^.

**Figure 6:**
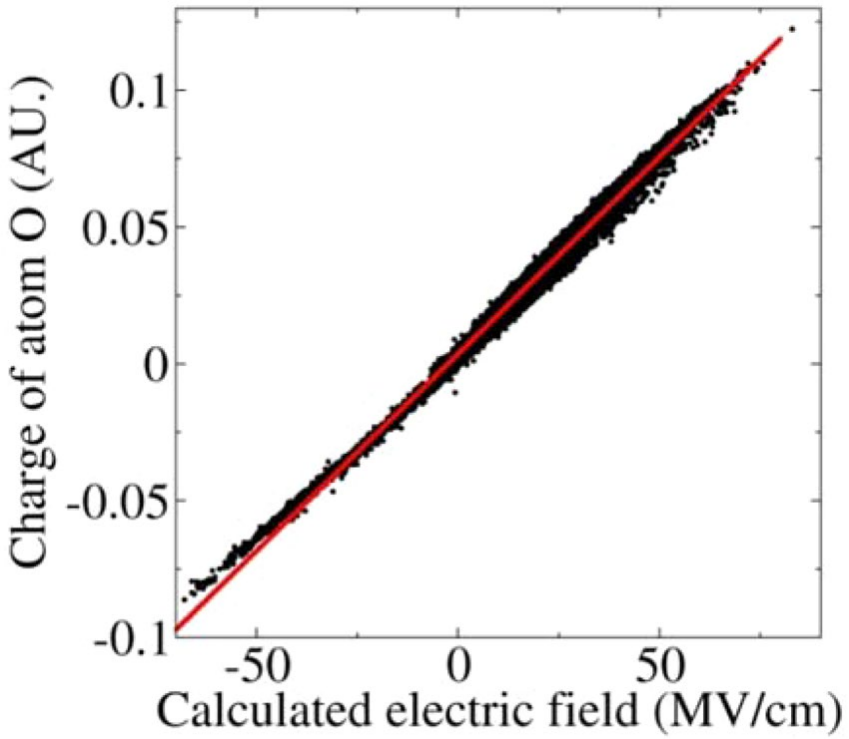
The obtained ESP charges in the O atom of the CO molecule derived from the QM/MM calculations in the MD simulation as a function of the electric fields along the CO bond, which exhibits a linear function relationship. The influence of the changes in the bond length of the CO was eliminated.

Corresponding with the quadratic function relationship between the interaction energies and the electric fields, the ESP-based atomic charges of the O atom of the CO molecule presented an exact linear relationship with the electric fields, as shown in Fig. 6. Similarly, the charge was also affected by the bond length (see panel A in Figure S3). Meanwhile, the fluctuation of the charges also had a linear relationship with bond length (see panel B in Figure S3). The positive direction of the electric field along the CO bond calculated using Eq. 4 pointed from the C toward the O. It can be seen from Fig. 6 that the O atom takes a positive and a negative charge in the positive and negative electric fields, respectively, which resulted in the CO molecule exhibiting C^−^O^+^ and C^+^O^−^ states. The linear relationship between the atomic charge and the electric fields may be utilized to introduce the polarization effect into the point charge model of classical force fields.

**Figure 7:**
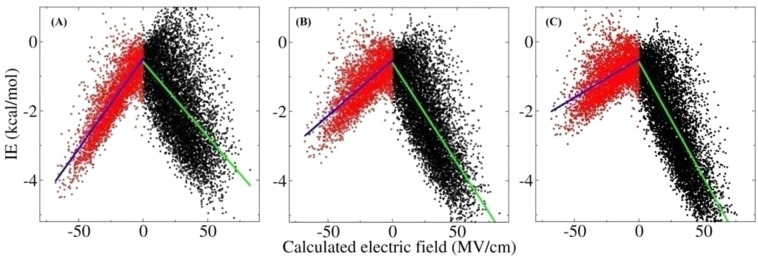
Distribution of the calculated interaction energies between the QM (CO molecule) and the MM (the surroundings) subsystems at the (**A**) HF/aug-cc-pVTZ/Amberff99SB, (**B**) B3LYP/aug-cc-pVTZ/Amberff99SB, and (**C**) MP2/aug-cc-pVTZ/Amberff99SB QM/MM levels as a function of the electric fields along the CO bond. The interaction energies exhibit a linear relationship with the electric fields. The green and blue lines represent the regression curves of the interaction energies relative to the positive and negative electric fields.

In actual QM/MM MD simulations, the interactions between the QM and MM subsystems undoubtedly contain multipole interactions that go beyond the classical point charge interactions, because these interactions are obtained using electronic structure methods. To statistically explore the physical fundamental of the two B states, we plotted the calculated QM/MM interactions at the HF/aug-cc-pVTZ/Amberff99SB, B3LYP/aug-cc-pVTZ/Amberff99SB, and MP2/aug-cc-pVTZ/Amberff99SB QM/MM levels as a function of the electric fields, with the results shown in Fig. 7. It can be seen from the figure that the obtained QM/MM electrostatic interactions of all the QM/MM models exhibited an approximate linear relationship relative to the positive or negative electric fields. Although different from the classical point charge interactions, the distribution of the QM/MM interactions in the positive and negative electric fields did not exhibit a symmetry that corresponded exactly with the different ratio of the two B states. To further understand the correlation between the distribution of the QM/MM interactions relative to the electric fields and the ratio of the two B states, the QM/MM interaction data were simply partitioned into two segments on the basis that the corresponding electric field was positive or negative and fitted with a linear function. The ratios of the two slopes of the fitted curves (*k*_1_ and *k*_2_ correspond to a positive and a negative electric field, respectively) for the results obtained at the HF/aug-cc-pVTZ/Amberff99SB, B3LYP/aug-cc-pVTZ/Amberff99SB, and MP2/aug-cc-pVTZ/Amberff99SB QM/MM levels are presented in Table 1. For comparison, the predicted B_1_/B_2_ ratio using the HF/aug-cc-pVTZ/Amberff99SB, B3LYP/aug-cc-pVTZ/Amberff99SB, and MP2/aug-cc-pVTZ/Amberff99SB QM/MM MD simulations are also presented in Table 1. The *k*_1_/*k*_2_ of the results for the HF/aug-cc-pVTZ/Amberff99SB, B3LYP/aug-cc-pVTZ/Amberff99SB, and MP2/aug-cc-pVTZ/Amberff99SB QM/MM calculations were 0.83, 1.80, and 3.02, respectively, which demonstrates a good correlation with the predicted B_1_/B_2_ ratios of 1.09, 1.54, and 3.45. Therefore, we can conclude that the physical fundamental that there are two B states with different proportions is a discrepancy in the QM interactions between the CO and the surroundings in antiparallel orientations.

**Table 1:** The obtained *B*_1_/*B*_2_ ratio from the QM/MM MD simulations at the HF/aug-cc-pVTZ/Amberff99SB, B3LYP/aug-cc-pVTZ/Amberff99SB, and MP2/aug-cc-pVTZ/Amberff99SB QM/MM levels. Here, *k*_1_/*k*_2_ is the ratio of the slope of regression curves for the QM/MM interaction energies relative to the electric fields, and *k*_1_ and *k*_2_ are the slopes that correspond to the green and blue lines in Fig. 7, respectively. ^a^Unsigned values are adopted.

Ratio	HF	B3LYP	MP2
*B*_1_/*B*_2_	1.09	1.54	3.45
^a^*k*_1_/*k*_2_	0.83	1.80	3.02

It is worth noting that the QM/MM interactions relative to the electric fields statistically presented a linear distribution as shown in Fig. 7, which indicates that the electrostatic interactions between the QM group and its surroundings can be described using a simple physical model of a dipole with constant moment under the action of an electric field. In the treatment, the interaction energy can be calculated using the formula $E=\overrightarrow{\mu }\cdot \overrightarrow{F}$, where $\overrightarrow{F}$ is the electric field, $\overrightarrow{\mu }$ is the dipole moment of the QM group (CO here) and $\overrightarrow{\mu }=q\overrightarrow{l}$, and $q\,{\rm{and}}\,\overrightarrow{l}$ are the electric-field-based equivalent atomic charge (EAC) and the bond length of the CO molecule, respectively. If the bond length adopted an approximate constant value of 1.14 ${\rm{\AA }}$, the obtained EAC will be C^−0.1638^O^0.1638^ (under the action of the positive electric field) and C^0.1979^O^−0.1979^ (under the action of the negative electric field) for the HF/aug-cc-pVTZ/Amberff99SB QM/MM calculations, C^−0.2196^O^0.2196^ (under the action of the positive electric field) and C^0.1222^O^−0.1222^ (under the action of the negative electric field) for the B3LYP/aug-cc-pVTZ/Amberff99SB QM/MM calculations, and C^−0.2541^O^0.2541^ (under the action of the positive electric field) and C^0.0843^O^−0.0843^ (under the action of the negative electric field) for the MP2/aug-cc-pVTZ/Amberff99SB QM/MM calculations. Here, it is worth noting that the EAC was statistically obtained unlike with the ESP-based charge model. The obtained *B*_1_/*B*_2_ ratio using the MP2/aug-cc-pVTZ/Amberff99SB QM/MM simulation was 3.45:1, which indicates that the C^−^O^+^ state played a dominant role in the interaction of the CO with the electric field. This result can explain the experimentally observed C^−^O^+^ polarity^96,97^.

**Figure 8:**
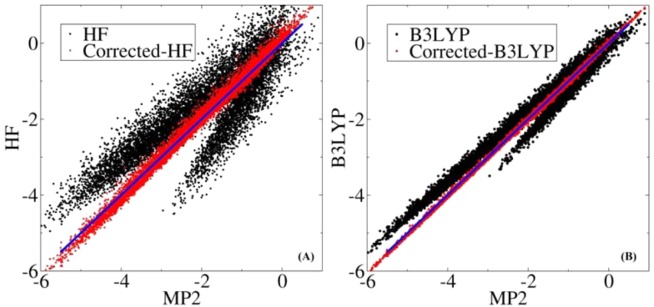
Correlation of the calculated QM/MM interaction energies (in kcal/mol) between the (**A**) HF/aug-cc-pVTZ/Amberff99SB, (**B**) B3LYP/aug-cc-pVTZ/Amberff99SB, and MP2/aug-cc-pVTZ/Amberff99SB QM/MM calculations. The corrected-HF and corrected-B3LYP mean that the obtained QM/MM interaction energies at the HF/aug-cc-pVTZ/Amberff99SB and B3LYP/aug-cc-pVTZ/Amberff99SB QM/MM levels were corrected with the obtained EFEAC charges. The blue line represents the strict correlation curve.

The obtained EACs for the CO were different across the various QM/MM calculations (HF, B3LYP, and MP2), and these were utilized to empirically introduce the electron correlation effects in the QM/MM MD simulation by correcting the QM/MM interactions using calculated interactions with the EACs. We tested the performance of this approach on the calculated QM/MM interaction energies at the HF/aug-cc-pVTZ/Amberff99SB and B3LYP/aug-cc-pVTZ/Amberff99SB QM/MM levels, with the interaction energies corrected by deducting the calculated interaction energies from the corresponding (HF or B3LYP QM/MM model) EACs and adding the interaction energies to the MP2 EACs. The correlation between the corrected HF and B3LYP interaction energies and the MP2 QM/MM calculations is shown in Fig. 8. For comparison, the correlation between the original HF and B3LYP QM/MM interaction energies and the MP2 QM/MM calculations is also plotted. It can be seen from Fig. 8 that the corrected interaction energies for both the HF and the B3LYP QM/MM calculations were in exact agreement with the MP2 QM/MM calculations, with a correlation coefficient (*R*^2^) of 0.9921 for the corrected HF and a *R*^2^ of 0.9995 for the corrected B3LYP. The mean unsigned error between them was only 0.101 kcal/mol for the corrected HF and 0.026 kcal/mol for the corrected B3LYP. Therefore, the approach is an accurate and effective method for introducing the electron correlation effects in QM/MM MD simulations. The physical foundation of the EACs is that the interactions of a chemical bond with the electric field possess an approximate linear relationship. Although this was only tested in terms of the sample MbCO system, many chemical bond groups (e.g., –OH, –NH, –SH, –CH, –C$\equiv$N, and >C$=$O) on various small molecules displayed a linear response to an electric field, which has been experimentally confirmed^23,25,98,99^. Therefore, the approach has a potential application for tackling various complicated QM/MM systems.

## Conclusions

In this work, QM/MM MD simulations of the ratio of the two B states of the MbCO system were performed to investigate how we can accurately model the QM/MM interactions with a QM/MM model. The calculated *B*_1_/*B*_2_ ratio was significantly sensitive to the size of the basis sets and was close to convergent after a basis set of aug-cc-pVTZ was reached. The introduction of both the polarization function and the diffuse function in the basis sets had a significant impact on the simulations. By comparing the obtained results at the HF/aug-cc-pVTZ/Amberff99SB, B3LYP/aug-cc-pVTZ/Amberff99SB, and MP2/aug-cc-pVTZ/Amberff99SB QM/MM levels, we demonstrated that the inclusion of the electron correlation effects is essential for accurately modeling the interactions between the QM and MM subsystems in QM/MM MD simulations.

The calculated electrostatic polarization energies based on the classical point charge model between the CO and the surroundings exhibited an exact quadratic function relationship with the electric fields along the CO bond. Meanwhile, the QM/MM interactions between the CO and the surroundings exhibited an asymmetric distribution in the positive and negative electric fields and presented a linear relationship with the electric fields. The QM/MM interactions could be described by a simple physical model of a dipole with constant moment under the action of the electric fields according to its statistical distribution relative to the electric fields. The treatment provides us with an accurate and effective approach to account for the electron correlation effects in QM/MM MD simulations. Given the limitation of our theoretical approach, the current study provided strong but incomplete evidence that there (statistically) exists an inevitable correlation between QM/MM interaction energies and the electric fields along the chemical bonds, which, nonetheless, could be utilized to account for the electron correlation effects in QM/MM studies.

## Supplementary information

Supplementary Information.
