# Supplementary material for: The Impact of Electron Correlation on Describing QM/MM Interactions in the Attendant Molecular Dynamics Simulations of CO in Myoglobin
Source: Sci Rep. 2020 May 22;10:8539. doi: 10.1038/s41598-020-65475-2 (PMC7244521; doi:10.1038/s41598-020-65475-2)
Supplement: Supplementary file 1 — Supplementary Information. [file 41598_2020_65475_MOESM1_ESM.pdf]

The Supplementary Materials for

The Impact of Electron Correlation on Describing QM/MM Interactions in the Attendant Molecular Dynamics Simulations of CO in Myoglobin

Xianwei Wang[1], Chenhui Lu[2], Maoyou Yang[3,4*]*

[1]*College of Science, Zhejiang University of Technology, Hangzhou, Zhejiang, 310023, China*

[2]*College of Mechanical Engineering, Shanghai University of Engineering Science, Shanghai 201620, China*

[3]*School of Electronic and information Engineering (Department of Physics), Qilu University of Technology (Shandong Academy of Sciences), Jinan, Shandong 250353, China*

[4]*State Key Laboratory of Precision Spectroscopy, East China Normal University, Sha nghai 200062, China*

* To whom correspondence should be addressed: xwwang@zjut.edu.cn and ymy@qlu.edu.cn.

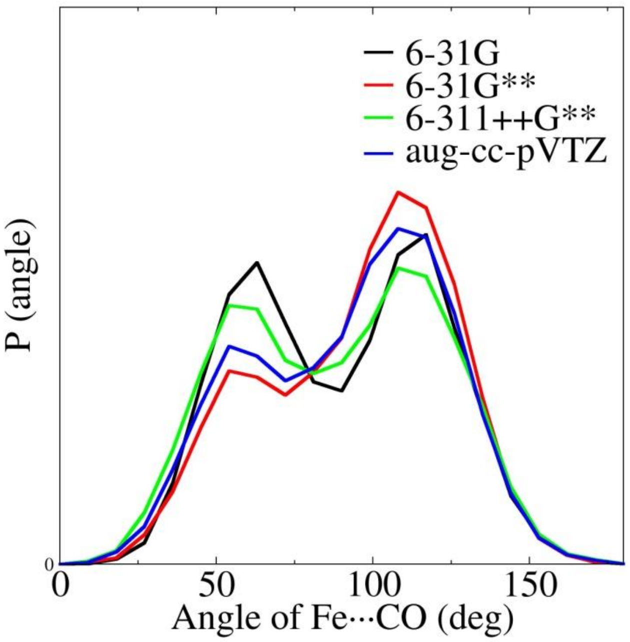

Figure S1. The distribution the Fe···C≡O angle for the QM/MM MD simulations at the B3LYP with basis sets of 6-311++G**, aug-cc-pVDZ and aug-cc-pVTZ QM/MM levels.

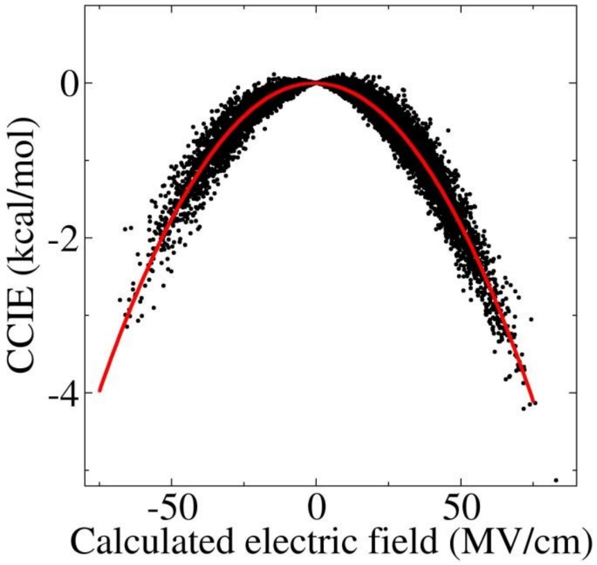

Figure S2. Distribution of the CCIEs between the QM (CO molecule) and the MM (the surroundings) as a function of the electric fields along the CO bond, which exhibits a quadratic function relationship. The influence on the CCIEs of changes in the bond length of the CO were included.

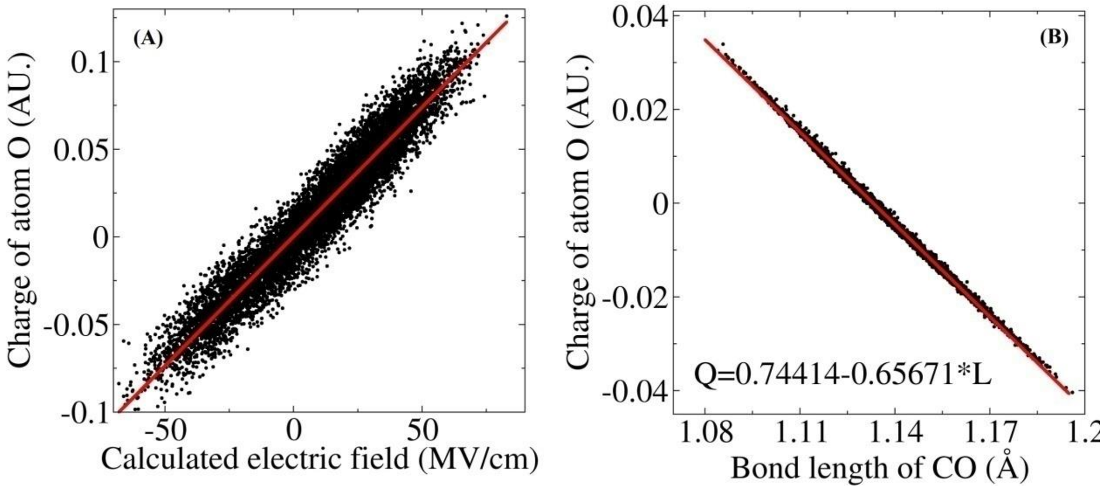

Figure S3. (A). The obtained ESP charges in the O atom of the CO molecule derived from the QM/MM calculations as a function of the electric fields along the CO bond, which exhibits a linear function relationship. The influence of the changes in the bond length of the CO was included. (B). The fluctuation of ESP charge of the O atom of the CO molecule as a function of the bond length of the CO, which exhibits a linear function relationship.
